# Developing Leadership Skills in Pharmacy Education

**DOI:** 10.1007/s40670-022-01532-x

**Published:** 2022-03-22

**Authors:** Raja Ali, Shaikha Jabor Alnaimi, Sara Abdulrhim, Fatima Mraiche

**Affiliations:** 1 College of Pharmacy, QU Health, Qatar University, Doha, Qatar; 2 Executive Pharmacy Office, Hamad Medical Corporation, Doha, Qatar

**Keywords:** Leadership, Manager, Pharmacy, Pharmacy education

## Abstract

Leadership could refer to holding official leadership positions or advocating for patients and the profession. Different pharmacy organizations recommend the inclusion of leadership development in pharmacy education and as such some pharmacy schools have introduced courses or various initiatives which support the development of leadership skills. In this commentary, we discuss means by which various pharmacy schools incorporate ways to develop leadership skills. This commentary also addresses the competencies used to assess leadership skills in pharmacy education.

**Supplementary Information:**

The online version contains supplementary material available at 10.1007/s40670-022-01532-x.

## Rationale for Leadership Development in Pharmacy Curriculum

Leadership has been defined as “a function of knowing yourself, having a vision that is well communicated, building trust among colleagues, and taking effective action to realize your own leadership potential” [1]. Leadership in healthcare involves providing direction, support, motivation, coordination, and advocating for patients to optimize their health outcomes [2]. A recent systematic review of studies assessing leadership in pharmacy education indicated that leadership is often defined as a way of influencing, motivating, and empowering others to help in achieving a common goal [3]. Other less commonly used definitions were those which defined a leader as a person who derives change and is recognized for their leadership roles either through holding a formal leadership position or by receiving a reward [3]. Despite the importance of leadership skills in healthcare, there is still no consensus regarding the meaning of leadership. Some relate it to holding leadership positions while others consider every healthcare professional as a leader regardless of their position. For the purpose of this commentary, leadership will refer to healthcare professionals being proactive in their roles and taking leadership roles regardless of their position.

A significant gap in pharmacy leadership within the following decade was predicted by White and colleagues who, in 2005, reported that most of the younger pharmacists, including pharmacy students, were not interested in holding leadership positions in the future [4]. However, over the next eight years, a slight improvement in the number of practitioners aiming to hold leadership positions was noticed [5]. Moreover, around 37% of the employers indicated that filling a vacant leadership position has become more difficult due to the lack of practitioners with leadership experience, lack of interest in these positions between current practitioners, and general belief that such positions are tough and stressful [5]. The lack of leadership skills in pharmacy students has also been recognized in an earlier study which identified the need to develop pharmacy students’ leadership skills to help them in driving change especially with the shift in pharmacy profession’s focus to patient-centred care [6]. The requirement for leadership skills development in pharmacy students is particularly important during difficult economical situations in which the efficient use of resources and practitioners’ skills becomes a paramount [7]. Effective leaders provide a vision for their team members and help organizations in achieving the goals set to meet that vision [8]. In addition, leaders motivate, inspire, and support their employees and this is important especially during times of crisis when people are often anxious and uncertain about the future. Collectively, these findings from the literature highlight the role of pharmacy schools in helping their students in discovering and developing their leadership skills by providing them with opportunities to engage in various leadership activities through collaboration with colleagues and by leading their teams to achieve a particular goal [9]. As a result, pharmacy schools have looked into ways to incorporate leadership skills’ development activities in their curricula.

Developing leadership skills in pharmacy graduates is recognized by various organizations and accreditation bodies. In 1982, the Argus Commission, which consists of the five most recent presidents of the American Association of Colleges of Pharmacy, highlighted in its report the importance of establishing a strong leadership education in pharmacy schools [7]. More recently, the Center for Advancement of Pharmacy Education (CAPE) in 2013 and the Association of Faculties of Pharmacy in Canada (AFPC) in 2017 advocated that leadership is one of the educational outcomes expected of pharmacy students [10, 11]. Accreditation bodies including the Accreditation Council for Pharmacy Education and the Canadian Council for Accreditation of Pharmacy Schools have identified the importance of including leadership developing content into pharmacy education in their standards [12, 13]. Further description of accreditation bodies’ standards related to leadership and management is provided in Supplementary Materials (Table S1).

According to CAPE, pharmacy graduates should demonstrate their ability to develop goals and effectively lead the team to achieve shared goals even if they are not officially occupying a leadership position [10]. There are several misconceptions associated with leadership among students one of which is the belief that leadership could only be practiced by holding a high position, also known as *positional leadership*. For example, a study that looked at predicting leadership gap in the pharmacy profession in the future reported that the younger pharmacists (including pharmacy students) were not interested in holding leadership positions in the future [4]. This could be partly explained by the fact that traditionally, pharmacy schools incorporated leadership development mainly into extracurricular activities and/or elective courses which contributed to the long-held belief that leadership is not an essential role for all pharmacist and that it is required to be possessed only by a few students who actively engage in such activities [14]. However, in order for the profession of pharmacy to benefit from the potentials of all its members, the concept of non-positional leadership must be emphasized. Sanders et al. defined non-positional leadership as “everyday influence processes by anyone in the organization derived from knowledge and the recognition for the need for a specific change” [15]. It is important to note that positional leadership is important in terms of maintaining authority and organizational hierarchy and is essential to facilitate the workflow and communication. However, it has been reported that non-positional leadership that is being demonstrated at all organizational levels is associated with more effective working relationships compared to traditional positional leadership [15]. This is especially true in complex and highly dynamic working environments such as the healthcare sector where waiting for the manager’s response might not be in the best interest of the patient and/or the organization. It has been reported that flexibility, innovation, and multidisciplinary decision-making is improved with the decentralization of authority [15]. Therefore, pharmacy curricula should aim to empower and train students to be able to identify opportunities for leadership in daily practice.

## Use of Leadership Competencies to Assess Leadership Skills in Pharmacy Education

In an attempt to provide standards for the assessment of content available to address leadership skills in pharmacy schools, several research groups have developed a number of leadership competencies [3]. In 2013, Janke et al. developed eleven leadership competencies to help curriculum committees in developing leadership content within their pharmacy curriculum [9]. Twenty-six leadership experts contributed to the development of these competencies following a thorough review process. The leadership competencies include elements related to leadership knowledge, personal leadership commitment, and leadership skills development. These leadership competencies were used to assess the leadership initiatives offered by 12 pharmacy schools in the United States (US) and it was found that the highest-rated competencies were “collaborate with others” and “describe the characteristics, behaviours, and practices of effective leaders”, while “develop knowledge of organizational culture” and “distinguish between leadership and management” were the lowest rated items [16]. Recently, Reed et al. extracted sixteen leadership competencies from studies which assessed leadership in pharmacy education and identified self-regulation, persuasion, strategic planning, and communication as the most commonly cited attributes in the literature [3]. Other leadership-related competencies including students’ knowledge, skills, abilities, and team orientation were also reported in some of the studies. Both competencies include elements that assess students’ knowledge of leadership, the skills and attitudes required for leaders, and efforts to improve leadership skills. A comparison between the two competency criteria in relation to the categories or the main themes assessed is provided in Table 1.

**Table 1:** Comparison of leadership skills’ development competencies for pharmacy education

Competency category	Janke et al. [9] criteria	Reed et al. [3] criteria
Knowledge	**Competency 1**: Explain the importance of leadership in pharmacy **Competency 2**: Recognize that leadership comes from those with and without titles **Competency 3**: Distinguish between leadership and management **Competency 4**: Describe the characteristics, behaviours, and practices of effective leaders **Competency 10**: Develop knowledge of organizational culture	1. Knowledge of leadership characteristics: leadership attitudes, skills, behaviours, abilities, and understanding the difference between leadership and management 2. Knowledge of pharmacy as a profession: history, role in the society, and issues that may shape the profession in the future
Skills, attitudes and qualities of leaders	**Competency 5**: Demonstrate self-awareness in leadership **Competency 8**: Collaborate with others	1. Social insight (emotional intelligence): ability to understand thoughts and feelings of other people and adjust one’s response accordingly 2. Effective communication (both spoken and written) 3. Perseverance 4. Negotiation and persuasion 5. Strategic planning 6. Relationship building 7. Decision-making 8. Personnel management: ability to align people with the roles in which they will develop and excel 9. Ethical orientation: a tendency to act in accordance with a set of moral and/or ethical standards 10. Service orientation: tendency to put the need of others above ones needs
Leadership skills development efforts	**Competency 6**: Engage in personal leadership development **Competency 7**: Develop a shared vision for an initiative or a project **Competency 9**: Lead members of a team **Competency 11**: Outline change process	1. Team orientation: a tendency to use collaborative and cooperative approach to decision-making 2. Learning orientation: a tendency to view experiences as opportunities to learn and develop

## Initiatives Used to Develop Leadership Skills in Pharmacy Students

The recognition of the need to advance leadership skills in pharmacy graduates resulted in the evaluation of existing content in different pharmacy programs, and many of the pharmacy schools in the US have since developed new leadership courses and programs [16, 17]. Other schools have responded by organizing retreats, speakers’ series programs, and other extracurricular activities [18–22].

A study which explored leadership development initiatives in different universities in the US reported that most schools (94 out 138) provided their students with leadership development opportunities [16], and were focused on providing students with the skills needed to derive a positive change in the practice and designed to ensure that students have a clear understanding of the overarching principle of leadership and its importance to the profession [9]. The range of activities provided by a selection of these schools is summarised in Table 2.

**Table 2:** Leadership development initiatives in pharmacy schools

Pharmacy school	Leadership development activities			
	**Type of activity**	**Academic year**	**Aim of the activity**	**Content**
College of Pharmacy in the University of Minnesota [18, 20]	**Elective course series** *The Leadership in Pharmacy Course* (2 credit hours over 2 semesters)	*Third year students*	• Educate students about leadership and the process of leading change • Encourage students to apply the skills learned in practice	• Didactic lectures • Experiential training • Self-directed learning
	**Extracurricular activity** *Retreat event*	Second, third, and fourth year students	Increase students’ awareness about leadership as a responsibility that is independent of the position and the need for commitment to excellence	• Self-reflection • Written assignments • Watching videos • Attending mini lectures • Group discussions • Team building activities
College of Pharmacy in the University of Oklahoma [23]	**Elective leadership development program** *Leadership Degree option (LDO)*	NR	• Increasing students’ self-awareness and responsibility to achieve goals • Improve students’ communication skills including their ability to empower others	• Five didactic courses offered over two academic years plus two Advanced Pharmacy Practice Experiences (APPE) • Various tests including Myers-Briggs Type Indicator (MBTI), Thomas Kilmann Instrument (TKI), Conflict Resolution Styles, Emotional Intelligence, and Strengths Finder
Regis University’s School of Pharmacy [19]	**Elective course** *Applied Leadership in Pharmacy Practice* (3 credit hours)	First and second year students	NR	• Assignments on self-discovery, development and application tools (e.g. Emotional and Social Competence Inventory and StrengthsFinder) • Writing a personal mission statement • Team building activities
Midwestern University’s College of Pharmacy [24]	**Elective leadership development program** (one and half academic year)	Selected students (mainly student organization officers)	NR	• Readings, self-assessments • large-group topic discussions and reflections written by students based on their application of the leadership concepts
College of Pharmacy at Purdue University [25]	**Mandatory session within a course**	First year students	NR	• Strengths assessment • Leadership videos • Guided discussions • An escape room exercise (Students in teams are required to solve five sequential puzzles within one hour)
College of Pharmacy & Health Science in Drake University [22]	**Course series** *Student Leadership Development series (SLDS)* (2 h/month for one year)	Students in leadership positions	• Help students in identifying their leadership style • Discussing problems encounter by student leader • Demonstrating their skills in creating relationships, assessing their ability to play the role of mentors • Assessing leadership skills application in practice	• Curricular activities • Co-curricular • Application of concepts through small group discussions with reflection • poster sessions
St. John Fisher College Wegmans School of Pharmacy [26]	**Co-curricular leadership program** *Student leadership challenge*		Enhancing students’ understanding of the leadership concepts and providing them with examples of leadership skills’ application in practice	-Three workshops each consisting of 30 min long lecture about leadership followed by participation in one of the students’ leadership challenge activities

*NR* not reported

At the College of Pharmacy, Qatar University, leadership content is distributed throughout 22 courses taught to students from year 1 to year 4. These courses vary in the level of leadership skills development from introductory level in the first two years to developing and mastery levels in courses offered in the latter two academic years. Our research team is currently planning to assess the effectiveness of this approach of leadership skills development through several courses in the pharmacy curriculum both among current students and alumni. A list of the courses addressing leadership in the College of Pharmacy at Qatar University with a description of these courses is provided in the Supplementary Material (Table S2).

Overall, it is clear that a wide variety of interventions has been introduced in pharmacy schools to develop the leadership skills of pharmacy students in practice. Encouragingly, students surveyed in some of the studies featured in Table 2 had a positive perspective on leadership programs and indicated that participation in these programs changed their view of leadership and helped in preparing them for applying the skills learned in practice [18–20, 22].

Additional interventions of leadership development in pharmacy education could be explored. Studies have also shown that leadership skills are improved by participation in peer-mentoring activities [20]. One study evaluated the effectiveness of a peer-mentoring program among occupational therapy students and identified a significant improvement in mentors’ leadership skills [21]. Surprisingly, this program also benefited mentees who were also taking a leadership course; mentees reported a greater improvement in their leadership skills as compared to students who only took the course [27]. Furthermore, involvement in professional and student organizations (e.g. American Pharmacists Association-Academy of Student Pharmacists) was found to enhance pharmacy students’ confidence, time management, and leadership skills [28].

## Conclusion

The importance of leadership development for pharmacy students has been highlighted by guiding organizations in pharmacy education, and various pharmacy schools who have developed programs and initiatives to address this skill. Several competencies associated with developing leadership skills have been documented in the literature, and a variety of initiatives including courses and extracurricular activities were shown to be effective in developing leadership skills. Further research is needed to assess the effects of leadership development content on pharmacy students’ leadership skills and performance in practice.

### Supplementary Information

Below is the link to the electronic supplementary material.

Supplementary file1 (DOCX 23 KB)

Supplementary file2 (DOCX 20 KB)

## Data Availability

Not applicable.
