# Correction to: Developing Leadership Skills in Pharmacy Education

**DOI:** 10.1007/s40670-023-01948-z

**Published:** 2023-11-23

**Authors:** Raja Ali, Shaikha Jabor Alnaimi, Sara Abdulrahim, Fatima Mraiche

**Affiliations:** 1 College of Pharmacy, QU Health, Qatar University, Doha, Qatar; 2 Executive Pharmacy Office, Hamad Medical Corporation, Doha, Qatar

**Correction to: Medical Science Educator (2022) 32:533–538** **https://doi.org/10.1007/s40670-022-01532-x**

The article “Developing Leadership Skills in Pharmacy Education” was originally published electronically on the publisher’s internet portal on 22 March 2022 with an error in the author group. The family name of the third author was misspelled as “Abdulrahim” instead of “Abdulrhim”.

The original article has been corrected.

