# Supplementary material for: Developing Leadership Skills in Pharmacy Education
Source: Med Sci Educ. 2022 Mar 22;32(2):533–8. doi: 10.1007/s40670-022-01532-x (PMC9054970; doi:10.1007/s40670-022-01532-x)
Supplement: Supplementary file 1 — Supplementary file1 (DOCX 23 KB) [file 40670_2022_1532_MOESM1_ESM.docx]

**Supplementary material:**

| Table S1. Leadership and Management Related Recommendations of Pharmacy Curriculum Organizations | | | |
| --- | --- | --- | --- |
| The accreditation standards | **Association of Faculties of Pharmacy of Canada AFPC (2017)^10^** | **ACPE (2016)^11^** | **CAPE (2013)^9^*** |
| Definitions | The AFPC does not differentiate between a leader and a manager’s role in their educational outcomes.   - *Definition:* As Leaders and Managers, pharmacy graduates engage with others to optimize the safety, effectiveness and efficiency of health care and contribute to a vision of a high-quality health care system. | - *Leadership definition:* The graduate is able to demonstrate responsibility for creating and achieving shared goals, regardless of position. - *Medication use systems management:* The graduate is able to manage patient healthcare needs using human, financial, technological, and physical resources to optimize the safety and efficacy of medication use systems. | - *Leadership definition:* Leadership involves inspiring others. It is a function of knowing yourself, creating a culture of trust and open communication, having a vision that is well communicated, empowering others, taking a broad view of situations, and forming strategic alliances. - *Management definition:* Identifying, implementing, and overseeing resources to effectively accomplish specific projects or processes. |
| Key competencies (what is expected from graduates in relation to leadership and management roles). | *LM1: Contribute to optimizing health care delivery and pharmacy services.*   - LM1.1 Work with others to apply quality improvement strategies and techniques to optimize pharmacy care. - LM1.2 Contribute to a culture of patient safety. - LM1.3 Confirm the quality, safety and integrity of products. - LM1.4 Use health informatics to improve the quality of care, manage resources and optimize patient safety.   *LM2: Contribute to the stewardship of resources in health care systems.*   - LM2.1 Apply evidence and management processes to achieve cost appropriate care. - LM2.2 Allocate health care resources for optimal patient care. - LM2.3 Contribute to the management of finances and health human resources in pharmacy practice settings.   *LM3: Demonstrate leadership skills*.   - LM3.1 Demonstrate leadership skills to enhance pharmacy practice and health care   *LM4: Demonstrate management skills.*   - M4.1 Work with others to apply the principles of effective management and supervision of health human resources and medication use systems. - LM4.2 Use effective strategies to manage and improve their own practice of pharmacy.   *PR3 (professional):* Committed to self-awareness in the management of personal and professional well-being.   - *PR3.1: Personal leadership:*   Set professional and personal goals, priorities and manage their time to balance patient care, workflow and practice requirements   - *PR3.2*   Examine, reflect upon and manage personal attributes (knowledge, skills, beliefs, biases, motivations, emotions) that could influence self-development and professional performance. | *Standard 4: Personal and Professional Development*  The program imparts to the graduate the knowledge, skills, abilities, behaviours, and attitudes necessary to demonstrate self-awareness, leadership, innovation and entrepreneurship, and professionalism.   - Display confidence in the patient care skills learned in pharmacy school. - Demonstrate professional behaviour (attitude, dress, appearance, etc.) in practice settings. - Embrace and advocate changes that improve patient care. |  |
| Means of addressing leadership/management roles in pharmacy schools |  | *Leadership:*   - Incorporating specific learning outcomes into the curriculum - Creating leadership-development programs for students - Supporting leadership development for faculty members so they may serve as role models for students and also advance the profession from the academic perspective   Management:   - Appendix 1 in the ACPE 2016 standards document provides a list of the required elements in the didactic content in the Doctor of Pharmacy program. One of the required courses is:   *Practice Management:*  After completion of the course the course the pharmacy students are expected to be able to apply the management principles learned including those related to operations, information, resources, personnel in addition to quality metrics in pharmacy practice.  The application of these principles is expected to improve patient care and service provision. | *Leadership:*  4.2. Leadership (Leader) - Demonstrate responsibility for creating and achieving shared goals, regardless of position  Examples of Learning Objectives:  4.2.1. Identify characteristics that reflect leadership versus management.  4.2.2. Identify the history (e.g., successes and challenges) of a team before implementing changes.  4.2.3. Develop relationships, value diverse opinions, and understand individual strengths and weaknesses to promote teamwork.  4.2.4. Persuasively communicate goals to the team to help build consensus.  4.2.5. Empower team members by actively listening, gathering input or feedback, and fostering collaboration.  Medication use systems management (Manager):  Examples of Learning Objectives:  2.2.1. Compare and contrast the components of typical medication use systems in different pharmacy practice settings.  2.2.2. Describe the role of the pharmacist in impacting the safety and efficacy of each component of a typical medication use system (i.e., procurement, storage, prescribing, transcription, dispensing, administration, monitoring, and documentation).  2.2.3. Utilize technology to optimize the medication use system.  2.2.4. Identify and utilize human, financial, and physical resources to optimize the medication use system.  2.2.5. Manage healthcare needs of patients during transitions of care.  2.2.6. Apply standards, guidelines, best practices, and established processes related to safe and effective medication use.  2.2.7. Utilize continuous quality improvement techniques in the medication use process. |

*CAPE Elaborates on the first 4 educational outcomes from the ACPE standards related to the knowledge, skills and attitudes that pharmacy graduates should possess.
