# Supplementary material for: Developing Leadership Skills in Pharmacy Education
Source: Med Sci Educ. 2022 Mar 22;32(2):533–8. doi: 10.1007/s40670-022-01532-x (PMC9054970; doi:10.1007/s40670-022-01532-x)
Supplement: Supplementary file 2 — Supplementary file2 (DOCX 20 KB) [file 40670_2022_1532_MOESM2_ESM.docx]

| Table S2. Leadership content in the Pharmacy Courses at Qatar University | | | |
| --- | --- | --- | --- |
| Level | Number of courses (%) | Course name | Course description |
| Introductory | 9 courses (40.91) | **Year 1 courses**  PHAR230  PHAR231  PHAR240  PHAR241  **Year 2 courses**  PHAR330  PHAR340  PHAR341  PHAR390  PHAR391 | *PHAR230 & PHAR231(Pharmacy &Healthcare I& II)*  This is a two-course series provided to first year pharmacy students. These courses are designed to introduce students to the role of the pharmacist within the health care system. In addition, through these courses, students are also introduced to current trends in pharmacy practice and the challenges faced in health care system. This series is also designed to begin developing competence in the practice of pharmacy.  *PHAR240, PHAR241, PHAR340, PHAR341, PHAR440, PHAR441 (Professional Skills I)*  This is a series of 6 courses designed to provide pharmacy students with the skills needed in practice. These courses are provided to pharmacy students throughout all the four pharmacy academic years. This series focuses on the development of knowledge and skills related to pharmaceutical care, medication prescribing and dispensing processes, and drug information resource retrieval and application in pharmacy practice. These courses also provide pharmacy students with the skills needed to interact with patients, customers, families and other healthcare providers.  *Structured Professional Skills Experience (SPEP):*  This program consists of six courses designed to provide students with a variety of practice-based opportunities to apply the knowledge and skills gained through campus-based learning. These opportunities will occur in selected hospital, community and clinic-based pharmacy practice sites and are structured around a number of formalized activities, each designed to lead to the attainment of specific learning objectives. These activities are supervised by pharmacy practitioners who serve as mentors, role models, trainers and assessors of student learning.  *PHAR390, PHAR391, PHAR490, PHAR491 & PHAR590 (Integrated case-based learning)*  This is a series of five courses which involves case studies aimed at integrating scientific and clinical concepts from across all courses in a problem-based learning environment. Patient case complexity increases across the sequentially delivered courses. Cases often focus on topics covered in integrated courses (i.e. pathophysiology, pharmacology and pharmacotherapy). Patient and disease management will occur in the context of a virtual health care environment.  *PHAR450 (Healthcare delivery systems):*  This course is intended to better prepare students to be knowledgeable about the various healthcare settings in which they may ultimately work. The specific goal of the course is to improve the students understanding of the development, organization, components and characteristics of contemporary health care systems.  *PHAR505 (Pharmacy Research evaluation and presentation skills):*  Pharmacy Research, Evaluation and Presentation Skills V (PHAR505) is fifth of six (PHAR305, PHAR306, PHAR405, PHAR406, PHAR505, PHAR506) courses designed to introduce the students to the detailed aspects of optimizing research design for clinical and basic research.  The topics covered in this course includes statistics, research design and developing critical appraisal of research skills.  *PHAR525 (Pharmacoeconomics):*  The PHAR525 course starts by providing brief understanding of the approach to resource allocation in relation to health sector. This course introduces students to techniques used to evaluate drug therapies from an economical aspect as clinicians and policy makers. The course also discusses some of the pharmacoepidemiology concepts and highlights different epidemiological study designs.  *PHAR535 (Pharmacy management)*  The Pharmacy Management course aims to provide comprehensive management overview in terms of concepts and techniques to students who are entering employment in any capacity within the field of pharmacy.  The course focuses on providing knowledge and skills related to areas of  This involves fostering the acquisition of knowledge and skills required to excel in the areas of entrepreneurship, resource management, business operations, value added services, marketing and risk management. In addition, the course aims to enhance students’  communication, critical thinking, problem solving, and team building skills. |
| Developing | 6 courses (27.27) | **Year 3 courses:**  PHAR430  PHAR440  PHAR441  PHAR450  PHAR490  **Year 4 courses:**  PHAR530 |  |
| Mastering | 7 courses (31.82) | **Year 3 courses:**  PHAR491  **courses:**  PHAR505  PHAR525  PHAR531  PHAR532  PHAR533  PHAR535 |  |
| Total | 22 courses (100) |  |  |
